# Advances in Nano-Drug Delivery for Tumor Microenvironment and Drug Resistance—Insights from the Special Issue “Nano-Drug Delivery Systems for Targeting the Tumor Microenvironment and Simultaneously Overcoming Drug Resistance Properties”

**DOI:** 10.3390/pharmaceutics17070942

**Published:** 2025-07-21

**Authors:** Patrícia M. A. Silva, Odília Queirós

**Affiliations:** 1 UNIPRO—Oral Pathology and Rehabilitation Research Unit, University Institute of Health Sciences (IUCS-CESPU), 4585-116 Gandra, Portugal; patricia.silva@cespu.pt; 2 Associate Laboratory i4HB—Institute for Health and Bioeconomy, University Institute of Health Sciences—CESPU, 4585-116 Gandra, Portugal; 3 UCIBIO—Applied Molecular Biosciences Unit, Translational Toxicology Research Laboratory, University Institute of Health Sciences (1H-TOXRUN, IUCS-CESPU), 4585-116 Gandra, Portugal

Cancer continues to pose a major global health burden [1], characterized by its biological complexity, high heterogeneity, and capacity to evade therapeutic interventions. Despite considerable advances in cancer research and the development of innovative treatment modalities, therapeutic resistance, whether intrinsic or acquired, continues to compromise treatment efficacy and patient outcomes [2]. Among the various forms of resistance, multidrug resistance (MDR) stands out as a major clinical hurdle. MDR is defined by the ability of cancer cells to evade the cytotoxic effects of multiple structurally and mechanistically distinct chemotherapeutic agents, often through diverse and overlapping molecular mechanisms. These may include altered drug transport, enhanced DNA repair, evasion of apoptosis, and, notably, the influence of the tumor microenvironment (TME) [3].

The TME, a dynamic and complex network of stromal cells, immune components, extracellular matrix, and abnormal vasculature, plays a critical role in modulating drug response and facilitating cancer cell survival. It contributes not only to physical and biochemical barriers that limit drug penetration, but also to signaling pathways that promote survival, angiogenesis, and immunosuppression. These factors are particularly pronounced in aggressive and recurrent malignancies such as pancreatic, triple-negative breast, and glioblastoma cancers [4,5].

In recent years, nanoscale drug delivery systems have emerged as promising tools to address both MDR and the challenges posed by the TME [6]. Compared to conventional formulations, nanocarriers offer several advantages, including improved solubility of hydrophobic drugs, protection of therapeutic agents from premature degradation, prolonged circulation time, and enhanced accumulation in tumor tissues via the enhanced permeability and retention (EPR) effect. Among these, endogenous stimulus-responsive nanocarriers, designed to react to specific tumor-associated stimuli such as pH, redox gradients, enzymes, or hypoxia, have demonstrated significant potential to enhance the specificity, accumulation, and therapeutic efficacy of anticancer drugs [7,8]. Additionally, multifunctional nanosystems capable of co-delivering chemotherapeutics with modulators of resistance pathways, or combining therapy with diagnostic imaging (theranostics), are opening new avenues for personalized and adaptive cancer treatment [9]. Despite encouraging preclinical results, the translation of these platforms into clinical practice remains a challenge, hindered by issues such as large-scale reproducibility, biocompatibility, and regulatory approval. Nonetheless, the ongoing evolution of nanotechnology continues to offer exciting prospects for overcoming the barriers imposed by MDR and the tumor microenvironment.

In the Special Issue “Nano-Drug Delivery Systems for Targeting the Tumor Micro-environment and Simultaneously Overcoming Drug Resistance Properties”, three original research articles and two review papers were published, highlighting innovative strategies in the development of nano-based delivery systems targeting the tumor microenvironment and overcoming drug resistance. Notably, one of the review articles was selected as an “Editor’s Choice”, highlighting its scientific relevance and valuable contribution to the field. Each contribution sheds light on innovative designs, mechanistic insights, and therapeutic potential across various cancer types.

Abobaker et al. developed a novel co-delivery nanoplatform combining iron oxide nanoparticles and PLGA (poly(lactic-co-glycolic acid)) for the encapsulation of curcumin (a natural anti-inflammatory and anticancer compound) and interferon-alpha (IFNα). The dual payload aims to enhance the therapeutic efficacy against A375 human melanoma cells. The authors reported significant synergistic cytotoxic effects, increased cellular uptake, and enhanced intracellular delivery. The platform leverages magnetic nanoparticle properties for potential image-guided therapy and exhibits controlled release kinetics that preserve drug bioactivity. This strategy exemplifies a multifunctional approach to overcoming drug resistance, which is especially relevant in solid tumors like melanoma where monotherapies often fail [Contribution 1].

Martino et al. explored the heterogeneous perfusion patterns within solid murine lung cancer tumors using computational and experimental techniques. The concept of perfusion anisotropy, where blood flow varies spatially within the tumor, was quantitatively evaluated to predict how it impacts the distribution and accumulation of nanotherapeutics. The study showed that poorly perfused regions, due to dysfunctional vasculature and interstitial pressure gradients, could hinder uniform drug delivery. These findings are critical for rational nanocarrier design, encouraging the tailoring of size, charge, and release mechanisms to overcome perfusion-limited delivery in solid tumors [Contribution 2].

Huang and colleagues developed a biomimetic delivery system using platelet membranes to camouflage and deliver a combination of diaminosuberoyl acid (DASA) and arsenic trioxide (ATO) into liver tumor tissues. The “Trojan Horse” design leverages the tumor-homing ability of platelets to evade immune clearance and actively target tumor endothelium. The formulation (DASA+ATO@PLT) demonstrated superior tumor penetration, improved drug accumulation, and effective remodeling of the tumor microenvironment to enhance chemotherapeutic efficacy. This approach represents a next-generation strategy that uses endogenous biological cues to overcome resistance and enhance targeted delivery in hepatocellular carcinoma [Contribution 3].

The reviews by Meng et al. and Cecchi et al. offer a conceptual and strategic synthesis of current advances, challenges, and future directions in the field. Meng et al. summarized the recent advancements in nanodrug delivery systems aimed at modulating the tumor microenvironment and circumventing multidrug resistance. The authors categorize nanocarriers into various responsive systems, such as those triggered by pH, redox potential, enzymes, and hypoxia, and examine how these stimuli-responsive systems enhance selectivity and reduce systemic toxicity. They also highlight the use of co-delivery platforms, gene-silencing technologies, and combination therapies that disrupt TME-mediated resistance. The review offers a forward-looking perspective on designing multifunctional nanocarriers for personalized, adaptive cancer treatment strategies [Contribution 4]. Cecchi et al. emphasized how nanoparticle-based strategies can augment conventional treatments, such as chemotherapy, radiation, and immunotherapy, by simultaneously targeting the tumor cells and their microenvironment. The review details how targeted delivery and TME reprogramming (e.g., normalizing vasculature, altering immune landscapes, or modulating extracellular pH) can improve therapeutic outcomes. The authors also assess the clinical translation challenges facing nanomedicines and call for interdisciplinary collaboration to advance next-generation delivery platforms. The work provides a clear, strategic roadmap for how nanotechnology can shift standard oncological paradigms toward precision-based and resistance-proof therapeutics [Contribution 5].

Collectively, these articles underscore the promise of integrating rationale design, biological insight, and translational ambition in the quest to overcome drug resistance through targeted modulation of the tumor microenvironment.

In summary, overcoming multidrug resistance mediated by the tumor microenvironment remains a critical challenge in cancer therapy. The nano-drug delivery strategies highlighted in this Special Issue demonstrate significant potential to enhance treatment specificity and efficacy through targeted, stimuli-responsive, and multifunctional designs. While clinical translation challenges persist, continued interdisciplinary efforts are essential to realize the full potential of nanomedicine in improving patient outcomes.
